# Vertical Dimension in Prosthodontics Theory and Practice (Part II): A Comprehensive Review of Vertical Dimension Determination in Prosthodontics From Classical Methods to Digital Innovation

**DOI:** 10.7759/cureus.93987

**Published:** 2025-10-06

**Authors:** Mostafa I Fayad, Rania Moussa, Nourhan A Ragheb, Yousra Ahmed, Sherif Sultan, Mahmoud R AbdulSalam, Hussein A Ismail, Mohamed O Elboraey, Mohammed H AbdElaziz, Mohamed A Helal

**Affiliations:** 1 College of Dentistry, Department of Substitutive Dental Science, Taibah University, Madinah, SAU; 2 Faculty of Dental Medicine, Al-Azhar University, Cairo, EGY; 3 Department of Prosthodontics, Faculty of Oral and Dental Medicine, Kafr El Sheikh University, Kafr El Sheikh, EGY; 4 Department of Prosthetic Dentistry, King Salman International University, South Sinai, EGY; 5 Department of Prosthodontics, Tanta University, Tanta, EGY; 6 Department of Prosthodontics, Faculty of Dentistry, Horus University, New Damietta, EGY; 7 Faculty of Oral and Dental Medicine and Surgery, Zagazig University, Zagazig, EGY; 8 Department of Preventive Dental Sciences, College of Dentistry, Taibah University, Madinah, SAU; 9 Department of Oral Medicine, Periodontology, Oral Diagnosis and Radiology, Faculty of Dentistry, Tanta University, Tanta, EGY; 10 Department of Substitutive Dental Science, College of Dentistry, Taibah University, Madinah, SAU; 11 Department of Fixed Prosthodontics, Faculty of Dental Medicine, Al-Azhar University, Cairo, EGY; 12 Faculty of Dental Medicine, Department of Prosthodontics, Al-Azhar University, Cairo, EGY

**Keywords:** 3d scanning, anthropometry, digital dentistry, facial measurements, occlusal vertical dimension, panoramic radiography, prosthodontics, vertical dimension

## Abstract

The determination of vertical dimension of occlusion (VDO) represents one of the most challenging and critical aspects of prosthodontic treatment, particularly in edentulous patients. Accurate establishment of VDO is essential for optimal function, esthetics, and patient comfort in prosthetic rehabilitation. This comprehensive literature review aims to analyze the current evidence regarding various methods for determining vertical dimension (VD) in prosthodontic practice, examining their reliability, clinical applicability, and evolution from traditional to modern digital approaches. A search was conducted across multiple databases, including PubMed/MEDLINE, Scopus, Web of Science, and Cochrane Library. The search strategy included terms related to VD, prosthodontics, complete dentures, and measurement techniques. A total of 1,259 articles were initially identified, with 903 remaining after duplicates were removed. Following screening and quality assessment, 79 high-quality studies were included in this comprehensive review. The analysis identified two main categories of VDO determination: (a) pre-extraction record methods and (b) post-extraction assessment methods, the latter encompassing anthropometric/biometric approaches, physiological and functional techniques, mechanical methods, measurement devices, radiographic or imaging modalities, and esthetic evaluations. While traditional techniques hold historical value, their reliability and reproducibility remain limited. In contrast, contemporary digital methods offer greater precision and patient comfort, though their use demands advanced equipment and practitioner training. While no single method has emerged as universally superior, the integration of multiple assessment techniques with digital technology offers enhanced accuracy and reproducibility. Contemporary evidence supports a multifactorial approach combining traditional clinical judgment with digital precision and novel radiographic formulas for optimal VDO determination.

## Introduction and background

The vertical dimension (VD) is defined as the measurement of the face between two selected anatomic or marked points (usually one on the tip of the nose and the other on the chin), in either the occlusal VD (OVD), with the teeth in maximal intercuspal position, or in the rest VD (RVD), with the mandible in a physiological rest position [1]. Accurate determination of VDO is fundamental to successful prosthodontic treatment, influencing facial esthetics, phonetics, masticatory function, and temporomandibular joint health [2,3]. The challenge of establishing appropriate VDO becomes particularly complex in edentulous patients, where natural reference points are absent [4].

The clinical significance of accurate VDO determination cannot be overstated. Inadequate VD can result in compromised esthetics, impaired function, temporomandibular disorders, and patient dissatisfaction [5,6]. Conversely, excessive VD may cause muscle fatigue, speech difficulties, bone resorption, and prosthetic instability [7,8]. Therefore, the selection and application of appropriate measurement techniques are crucial for optimal treatment outcomes [9].

Historical perspectives on VDO determination date back to the early 18th century. Then, the pioneering work by Niswonger [10] established the concept of physiologic rest position. Since then, numerous methods have been developed, ranging from simple mechanical measurements to sophisticated digital analysis [9,11]. The evolution of these techniques reflects advances in dental materials, technology, and understanding of orofacial anatomy and physiology [12].

Early foundations (before and early 19th century)

During the 15th century, Leonardo da Vinci proposed simple facial ratios for artistic representation. These proportional concepts were later adapted for application in complete denture construction [13].

The concept of VD in prosthodontics originated in the early period of complete denture fabrication. In 1771, Hunter was one of the earliest clinicians who published observations related to facial proportions and the spatial relationships between opposing jaws. Hunter’s early insights into the physiological rest position of the mandible are considered a foundational concept for understanding VD in complete denture fabrication [14]. However, there was no specific method for determining VD during this early period.

In 1887, Ivy adapted the proportional concepts of Leonardo da Vinci for application in complete denture construction. Ivy suggested that the human face could be divided into four equal parts, and these divisions were used as guidelines during prosthodontic procedures [13].

19th century - early scientific methods

As prosthodontics began to formalize towards the late 19th and early 20th centuries, practitioners emphasized achieving vertical jaw relationships in denture construction. However, these methods were primarily empirical, based on aesthetics and comfort rather than rigorous scientific validation. While specific early clinical journal citations are scarce, this trajectory is highlighted in modern literature reviews that underscore the historical reliance on trial-and-error techniques [15].

In 1906, Wallish provided one of the first clear definitions of the physiological rest position of the mandible: a position where all muscle action is eliminated, the mandible is passively suspended, and teeth do not contact-an important conceptual advance [14].

In the 1930s, Niswonger [5] introduced the concept of using the physiologic rest position as a reference for establishing the VD. This was a major departure from earlier fixed reference points to new physiologic reference positions. According to Niswonger, the difference between the rest VD and the occlusal VD, called the freeway space, was found to be approximately 2-4 mm, a concept that is still adopted in the modern era [14].

In 1933, Goodfriend refined Ivy’s approach, emphasizing that the distance from the pupil to the rima oris should equal the distance from the base of the nose to the chin [13].

In 1935, Willis supported Goodfriend’s observations and introduced a specialized instrument - the Willis Gauge - "bite-gauge" and suggested the concept of "harmonic faces," aiming to establish facial harmony by ensuring the lower third of the face was proportional to the upper and middle thirds. Later, in 1953, Fenn recommended using the distance from the outer canthus of the eye to the angle of the mouth as an additional guide for establishing the correct VD of occlusion [13].

Mid-20th century - functional and physiological approaches

The mid-20th century marked notable progress in the development of a scientific approach to determining the VD. McGee [16] used the facial measurements in determining VD, providing relatively standardized measurements that were widely adopted in clinical practice.

Silverman [17] further developed phonetic methods, in particular the closest speaking space concept. This suggested that, during the pronunciation of certain fricative sounds, maintaining a slight interocclusal distance might be significant, indicating that the VD could be related to the functional requirements of speech [18].

Cephalometric analysis also became popular in this era as it was a more scientific and accurate method of assessing craniofacial relationships. This radiographic approach offered unprecedented precision in measuring facial dimensions and establishing reproducible reference points [19,20].

Late 20th century - neuromuscular and temporomandibular joint (TMJ) focus

In the late 20th century, emphasis shifted to understanding the roles of TMJ dynamics and muscle activity in VD determination. Techniques such as electromyography (EMG) recordings introduced more objective approaches [21].

Early 21st century

There has been a transition towards more evidence-based approaches and the incorporation of digital technologies in VD determination. The development of VD concepts and methods reflects the broader evolution of prosthodontics as a discipline, from empirical approaches mainly based on clinical experience to evidence-based approaches informed by scientific research and technological innovation [22,23].

While Part I of this series outlined the conceptual evolution of VD, the need for a subsequent review arises from the growing number of methods developed to establish this parameter in clinical practice.

Although numerous classical methods - such as facial and anthropometric measurements, phonetic assessments, and swallowing techniques - have been described, previous reviews provide inadequate coverage of contemporary digital technologies. Over the past decade, significant progress has been made with imaging modalities, computer-aided design and manufacturing (CAD/CAM), three-dimensional facial scanning, cone-beam computed tomography (CBCT), and, more recently, artificial intelligence-based diagnostic tools. Despite their growing clinical application, these digital approaches are often only briefly discussed or entirely omitted in earlier reviews, leaving a gap in understanding their utility, comparative effectiveness, and integration with traditional techniques. This review aims to bridge these gaps by examining conventional approaches, evaluating recent digital innovations, and outlining future directions in the determination of VD in prosthodontics.

## Review

Methodology

This comprehensive literature review was conducted in accordance with established guidelines for narrative reviews in dentistry. A systematic search strategy was employed across multiple electronic databases, including PubMed/MEDLINE, Scopus, Web of Science, and Cochrane Library. The search encompasses both historical foundational works and contemporary research.

The search strategy utilized a combination of Medical Subject Headings (MeSH) terms and free-text keywords, including "vertical dimension", "occlusal vertical dimension", "prosthodontics", "complete dentures", "facial height", "mandibular rest position", "cephalometric analysis", "phonetic method", and "digital dentistry". Boolean operators (AND, OR) were used to combine search terms effectively.

The inclusion criteria comprised peer-reviewed articles in English, studies involving human subjects, research focusing on VDO determination methods, both clinical and laboratory studies, and articles with clear methodology and results. Exclusion criteria included articles without a clear methodology, non-English publications without available translations, and studies that focused solely on pediatric populations.

The initial search yielded 1,259 articles. After removing duplicates (n = 356), 903 articles underwent title and abstract screening. Following full-text review and quality assessment using modified Newcastle-Ottawa scale criteria, 79 high-quality studies were included in the final analysis. This systematic evaluation facilitated the identification of studies with sufficient quality to address the research objectives.

The analysis of the included studies revealed a broad spectrum of perspectives tracing the conceptual and clinical evolution of VD in prosthodontics. These works collectively illustrate how early artistic and anatomical observations gradually informed dental practice, shaping the foundations of current approaches. To appreciate this progression, it is essential to begin with the earliest documented notions of VD in the historical record.

Having identified and appraised the relevant literature, this review is organized to present the historical evolution of the VD in prosthodontics. The discussion will focus on the principal methods of VD determination as described in the literature. These methods are generally classified into two main groups - pre-extraction and post-extraction approaches - with the latter further subdivided into six categories: anthropometric/biometric methods, physiological and functional methods, mechanical methods, measurement devices, radiographic and imaging methods, aesthetic approaches, and digital technologies.

Discussion

Vertical Dimension Assessment Methods and Techniques

A. Pre-extraction records methods

Pre-extraction records represent the most reliable method for determining VD when available [22]. This approach involves measuring and documenting the existing VD before tooth extraction, particularly when patients have maintained acceptable function and esthetics with their natural dentition [24].

When pre-extraction diagnostic casts are available, Heintz et al. [25] recommended utilizing the recorded VD and occlusal relationships as a reference for fabricating subsequent dentures. Similarly, several authors have described the use of specific oral landmarks to approximate vertical height. For instance, Smith [26] proposed the placement of tattoo dots on the attached gingiva of both the maxillary and mandibular arches to serve as permanent markers. Nevertheless, this method presents notable drawbacks: the resilience of soft tissues can compromise measurement accuracy, and clinicians must also consider the wide inter-individual variation that limits the reliability of such landmarks [3,26].

The interfrenal distance, or the space between the maxillary and mandibular labial frena when the teeth are in occlusion, can be used as a pre-extraction record to help determine the occlusal VD (OVD) for a complete denture. This measurement is typically performed with a pair of dividers. However, the reliability of this method is compromised if the frenal attachments have been surgically altered or if extensive alveolar ridge resorption has occurred, as these changes can displace the frenum and invalidate the original measurement [3].

The OVD can be assessed using profile tracing of the lower third of the face. Several methods exist for generating such a tracing template. A more straightforward option is the Sorenson profile scale, which records the distance between two predetermined facial points when the teeth are in maximal intercuspation [27].

Other devices, including the Dakometer and Willis gauge, may also be employed to measure facial tissue dimensions. However, these soft tissue-based techniques are inherently limited, as tissue resilience and operator variability may introduce distortion and compromise accuracy [28].

The cardboard profile record is a pre-extraction method for documenting a patient's natural facial contour and OVD. The technique involves creating a cardboard template that accurately traces the patient's median facial profile while their natural teeth are in occlusion. This record is then archived to serve as a reference guide during complete denture fabrication. A significant drawback is that it captures a static moment in time, and facial contours can change over time due to aging, skin laxity, and muscle changes, potentially affecting the long-term accuracy of the record [26].

A facial template, or mask, could be used to capture the original facial contour, jaw relationship, and tooth characteristics. In addition to preserving the OVD, a key advantage of the template is its ability to record the natural contours of the lips, which provides a crucial guide for accurately positioning the anterior teeth. However, the use of a template has been reported to exhibit potential inaccuracies of 2 mm or more, as the measurements are based on soft tissue, which can be affected by the resilience of the skin [3].

The cephalometric approach provides a more objective method. A cephalometric radiograph is first obtained before extractions, followed by another radiograph taken with occlusal rims after extractions. By comparing these pre- and post-extraction images, clinicians can accurately adjust the occlusal rims to establish the correct VD [29].

The pre-extraction photographs could also be used to serve as a valuable reference, enabling clinicians to restore the patient’s VD during denture fabrication [24,27,30].

More recently, digital facial scanning has emerged as a modern alternative for evaluating facial proportions and vertical height. This method offers notable advantages, including simplicity, rapid image acquisition, and the ability to store records digitally rather than physically. In addition, dedicated software allows precise visualization and accurate quantification of distances between facial landmarks, enhancing both diagnostic and treatment accuracy [24,31].

B. Post-extraction assessment methods

1. Anthropometric/Biometric Methods

They utilize correlations with facial and body measurements of anatomical landmarks and proportional relationships to estimate the appropriate VD. The classical approach involves dividing the face into equal thirds, with the lower facial height corresponding to the distance from the base of the nose to the chin [32].

McGee [16] suggested correlations between facial measurements and VD. More recent studies have explored these relationships with greater scientific rigor. Singh et al. [32] investigated correlations between VD at occlusion and 13 anthropometric measurements. They found that twice the length of the eye and the distance between the tip of the thumb and the tip of the index finger were closest to the VD at occlusion in male patients, while the vertical distance from the pupil to the corner of the mouth and the vertical height of the ear were closest in female patients.

Emam [33] found significant correlations between the nose-to-chin distance and other facial measurements, particularly the distance from the pupil to the corner of the mouth in edentulous patients and the distance from the outer canthus of one eye to the inner canthus of the other in dentate subjects.

Dhoot et al. [34] found a positive relationship between VDO and the height of the external Ear. Therefore, this method could be useful in daily practice to determine VDO.

The Knebelman craniometric method could be used for the estimation of the vertical dimension of occlusion (OVD), ensuring occlusal harmony and balanced occlusion. This approach measures the eye-ear distance, specifically the distance between the anterior wall of the external auditory canal and the outer canthus of the eye [35].

Avila-Vásquez et al. [36] conducted a cross-sectional study involving 200 patients to compare the anthropometric and Knebelman craniometric methods for VDO estimation. While both produced statistically equivalent mean values, Knebelman’s method exhibited significantly less measurement variability (p < 0.05), indicating superior reliability across different facial biotypes.

A recent study has shown a good concordance between the Knebelman method and other techniques, such as functional swallowing [37]. The technique has been described as an inexpensive, straightforward, and non-invasive method for determining OVD. However, the development of predictive models that utilize the right or left eye-to-ear distance for determining VDO in both dentate and edentulous individuals is influenced by factors such as age, gender, ethnicity, and facial type [35].

Bhat et al. [38] carried out a clinical study to investigate the relationship between intercondylar distance and OVD in dentate individuals. Their findings revealed a significant positive correlation between the two measurements, indicating that OVD can be estimated from intercondylar distance through a regression model.

The concept of divine proportion (golden ratio) has been applied to facial aesthetics and, by extension, to VD determination. This approach suggests that certain proportions in the face, when in harmony with the golden ratio (approximately 1:1.618), result in aesthetically pleasing outcomes. Ward [39] discussed the application of the golden proportion to dental aesthetics, including its relevance to OVD. This approach considers the OVD in the context of overall facial harmony rather than as an isolated measurement.

Some anthropometric approaches extend beyond facial measurements to include correlations with body dimensions. Ladda et al. [40] investigated the relationship between OVD and interpupillary distance (IPD), finding significant correlations that could be used as guides in edentulous patients.

Bacali et al. [41] found a high statistical correlation between OVD and the palm width measured at the fingers’ base. Additionally, statistical correlations were found between the VDO and the middle finger length. They concluded that a simple formula using finger length/palm width can be used for a rapid VDO determination.

In a cross-sectional study of 250 dentate individuals, Hussain et al. [42] demonstrated a strong positive correlation between index finger length and OVD.

The advantage of these anthropometric methods is their non-invasive nature and the ability to use multiple reference points for cross-verification. However, variations across gender, ethnicity, and race limit the universal applicability of specific measurements [32].

While this method is simple and widely used, its reliability is questionable. The soft tissues can be easily displaced, leading to inaccurate measurements [13].

2. Physiological and Functional Methods

2.1 Physiological Rest Position

Physiological methods for VDO determination are based on the principle that the mandible assumes a consistent rest position when the facial muscles are in equilibrium. The freeway space concept, representing the distance between maxillary and mandibular teeth when the mandible is in rest position, forms the foundation of this approach [43,44].

The rest position identification technique involves asking patients to relax their facial muscles; with the head held upright, the lips are allowed to contact lightly, and the space between facial reference points is measured. From this, the freeway space is subtracted to give the OVD [18]. However, the rest position is not as stable as once thought and can be influenced by factors such as posture, emotional state, time of day, and the presence of pain [14].

2.2 Swallowing Technique

The swallowing method utilized the physiologic activity of swallowing as the means of evaluating the VD. During the swallow, the mandible is thought to posture near an appropriate OVD. Softened wax rims were placed into the mouth, and the patient repeatedly swallowed, with the wax being cut back until the patient could swallow without difficulty [45,46].

The advantage of this technique is its functional basis, as it relates the VD to a natural physiologic activity. However, the reliability of this method can be affected by variations in swallowing patterns and the presence of abnormal swallowing habits [47].

2.3 Phonetic Methods

Phonetic methods utilize speech sounds to determine the proper VD. Gillis [48] was among the first to suggest that certain speech sounds could be used for this purpose. Silverman [17] further developed this approach with the concept of the closest speaking space.

The phonetic method typically involves having the patient pronounce sibilant sounds (such as "s" or "ch") or words containing these sounds (e.g., "sixty-six" or "Mississippi"). During these pronunciations, there should be a minimal clearance (about 1-2 mm) between the anterior teeth or occlusal rims.

Pound [49] emphasized the importance of phonetics in determining not only the VD but also the position of anterior teeth. This method has gained widespread acceptance due to its functional basis and relative simplicity.

Igić et al. [50] found that an approximate value of interocclusal space during vowel pronunciation “O” of 5.5 mm and 7.5 mm of vowel “E” pronunciation can be used to determine OVD in both genders.

The phonetic procedures are still one of the most reliable clinical techniques for assessing VD, indicating their ongoing significance in modern practice. However, with the presence of linguistic and phonological variations as well as different articulations of the consonant and vowel letters among different populations, it might be quite difficult to generalize this technique for a wide population [3].

2.4 Neuromuscular Perception (Tactile Sense)

This method relies on the patient's neuromuscular perception and ability to sense optimal VD through proprioceptive feedback. While this approach has shown promise in reducing adaptation time, it requires patient cooperation and may be influenced by individual variations in proprioceptive ability. Additionally, the presence of the screw-jack in the patient’s mouth may affect the accuracy of the oral perception and may restrict the freedom of the tongue [3].

Lytle [51] adopted a more refined technique using a central bearing device fixed to the upper and lower occlusion rims. The patient is asked to close "just as he had his own natural teeth present in his mouth," requiring the patient to recognize this position through tactile sense.

2.5 Mastication-Based Assessments

This approach recognizes that the optimal VD should facilitate efficient and comfortable mastication. A technique was described that involved having patients chew on wax rims, gradually adjusting the VD until optimal chewing efficiency was achieved. This method acknowledges the functional nature of VD but can be subjective and time-consuming [52].

3. Mechanical Methods

3.1 Paralleling the Ridges

Wright [53] described the parallelism of the ridges technique, where the maxillary and mandibular ridges are thought to be parallel to one another when the jaws are in the correct VD.

However, this method suffers from problems related to the differing and variable resorption patterns of edentulous ridges and lacks a scientific basis. The timing and extent of tooth loss also vary widely among patients, further invalidating the assumption of parallelism [54].

3.2 Patient's Existing Complete Denture

Massad et al. [55] reported a technique to use a patient's existing complete denture to determine the VD of occlusion. The advantage of this approach is that it utilizes the patient's adaptation to their existing prosthesis, which can reduce the adaptation time to the new restoration.

However, this method only works under the assumption that the existing prosthesis is of an acceptable VD, which is not always the case. Additionally, pre-extraction records are often not available, especially in long-term edentulous patients [15].

4. Measurement Devices

4.1 Mechanical Devices

The Willis gauge is specifically designed to measure facial dimensions related to the VD. It measures the distance from the base of the nose (subnasion) to the bottom of the chin (gnathion) when the teeth are in occlusion. It remains one of the most widely used tools for VD determination due to its simplicity and ease of use.

However, Tina-Olaivar et al. [56] conducted a study on a Filipino sample; their findings demonstrated that the upper facial measurement exceeded the lower facial measurement by approximately 3 cm, revealing that the underlying assumption of facial height equivalence in the Willis method does not hold true in this population.

The calibrated calipers or dividers provide another mechanical means of measuring VD. Unlike the Willis gauge, calipers typically measure the distance between points on the nasal tip and chin. Singh et al. [32] noted that this method is influenced by soft tissue compression in the region of the skin markers. The advantage of calipers is their precision in measurement, but they share similar limitations with the Willis gauge regarding soft tissue compression and the need for consistent reference points [57]. Modified digital Vernier calipers provide high-precision measurements of various facial or intraoral distances to help estimate VDO.

The Knebelman craniometer is a specialized device used for determining the OVD, particularly for edentulous patients. It was developed and patented by Dr. Stanley Knebelman based on the principle of craniometry, which posits that a predictable, proportional relationship exists between certain craniofacial features [37].

The Boos bimeter is a mechanical instrument developed to establish the OVD according to the “power point” theory, which suggests that the maximum biting force is achieved at the appropriate VD. It incorporates a pressure gauge and a central bearing point that registers the force generated by the patient when biting on occlusal rims at varying degrees of jaw separation [3]. However, the bimeter has been criticized due to the influence of pain and patient apprehension on closing force. Comparisons between bimeter readings and those obtained through clinical assessment and electromyography revealed that its use often resulted in exaggerated VD values [58].

A novel device, the Precise Jaw Relation Recorder, has been developed to simultaneously register the occlusal plane, VD, and centric jaw relation in both edentulous and dentulous patients. It comprises vertical and horizontal arms, with the vertical arm carrying an upper component that aligns the occlusal plane parallel to the ala-tragus line. The lower component incorporates an adjustable bite fork to capture the centric mandibular position, while nasal and chin pointers are used to measure the patient’s vertical facial dimension. Additionally, the device includes a transfer assembly that enables the recorded jaw relations to be accurately transferred from the patient to an articulator [59].

4.2 Electronic and Advanced Devices

Electromyography (EMG) and jaw trackers can be used to provide more objective and precise measurements, particularly in complex cases or for research purposes. EMG measures the electrical activity of jaw muscles, such as the temporalis and masseter. When combined with jaw-tracking technology, this can objectively determine the neuromuscular rest position, which is used to calculate the VDO [60].

4.3 Transcutaneous Electrical Neural Stimulation (TENS)

The use of TENS in dentistry is a well-documented technique, primarily within the field of neuromuscular dentistry. It provides a way to physiologically relax the jaw muscles, which could allow for a more accurate and reproducible determination of the jaw's rest position, an essential step in establishing the correct VDO during dental treatment [61,62].

Kinesiographic analysis of mandibular movements using the K7 Evaluation System can trace mandibular movement during swallowing or at different VDOs, providing detailed trajectory data to aid in determining the correct VD [45].

5. Radiographic/Imaging Methods

Radiographic techniques for VDO determination offer objective, measurable approaches that can be documented and reproduced. Cephalometric analysis provides a reliable and objective method for evaluating craniofacial relationships, including the VD [29].

Advances in digital imaging have expanded the possibilities for VD assessment. Digital cephalometry provides better image quality and allows for computerized cephalometric analysis.

A recent innovative radiographic method for VD determination was developed by Fayad et al. [63]. They implemented a new formula to estimate the VD of occlusion from a panoramic radiograph using the distance between the mental foramina as a reference measurement.

One of the recent advancements in this category is 3D facial scanning, which captures detailed surface topography of the face. This technology allows for precise measurements of facial dimensions and can be particularly valuable in tracking changes in VD over time [23].

6. Aesthetic Approaches

Esthetic approaches prioritize restoring the patient's natural facial proportions - especially in the lower third of the face - rather than relying solely on mechanical or physiological measurements. These methods emphasize creating harmonious facial balance, taking factors such as orofacial symmetry and volume into account to guide the determination of VDO. For instance, adjusting the OVD via prosthetic rehabilitation can enhance the aesthetics of the lower facial third, improve soft-tissue symmetry, and even reduce the need for additional fillers or invasive facial procedures [64].

Facial appearance and harmony assessment involves consideration of aspects such as facial thirds proportion, lip support, and the lip-teeth relationship. The relationship between the VD and lip support is of special interest in the edentulous patient [65].

Digital Smile line analysis is an emerging topic in esthetic analysis with regard to the VD. The VD affects the position of the incisal edges relative to the lower lip in smiling and can have a major effect on esthetics. This analysis involves assessing the relationship between the incisal edges of the maxillary anterior teeth and the curvature of the lower lip during smiling. At an optimal VD, the incisal edges harmonize with the lower lip contour, producing a natural and esthetically pleasing smile. A reduction in VD often results in excessive tooth display loss, flattening of the smile arc, and deepening of perioral wrinkles, thereby creating an aged appearance [66].

7. Recent Advances - Digital Methods and Technologies

7.1 3D Imaging Techniques

The digital impressions, combined with CBCT data, allow for the 3D visualization of patient conditions and detailed treatment planning [67]. Virtual articulators now integrate intraoral scans, facial scans, and CBCT to enhance treatment predictability by reproducing the relationship between the jaws in a virtual environment [68].

7.2 Digital Wax-Ups and Virtual Articulators

This technology allows for precise adjustments and planning before physical fabrication. Studies have explored the use of digital wax-up training systems, utilizing CAD software, to enhance dental prosthesis design [69].

7.3 Software Applications for Analyzing Vertical Dimension

Specialized software applications play a critical role in the digital determination of VD. Computer-aided design software, such as Exocad dentalCAD, enables the reproduction of positional relationships with altered VD, allowing clinicians to select an optimal VD based on factors such as facial height and freeway space [70]. Digital software also significantly minimizes manual errors and provides precise measurements, ultimately saving clinicians' time and enhancing the sophistication of clinical work [71].

7.4 Use of AI and Machine Learning in Assessment

AI and machine learning are increasingly used for automated diagnostics, predictive measures, and classification or identification tools [72]. AI models have been developed to assist in tasks such as designing removable partial dentures, classifying partially edentulous arches, predicting functional outcomes, and even designing fixed denture prostheses that mimic natural tooth morphology [73]. These AI-driven methodologies are also being integrated into virtual articulator systems, further refining the precision of VD assessment [68].

Comparative Analysis: Traditional Versus Digital Methods

The digital methods have demonstrated comparable, and sometimes superior, accuracy to conventional techniques. For instance, the digital removable dentures have been shown to have superior occlusal accuracy compared to conventionally made dentures [74].

The accuracy of virtual occlusal records obtained with intraoral scanners shows promising results and high agreement with traditional methods, although factors such as scanner brand, imaging technology, scan quality, and software algorithms can influence this accuracy [75]. However, achieving high accuracy in fully edentulous scans remains a challenge due to the lack of sufficient reference points [76,77].

The digital workflows streamline processes, potentially reducing chair time and overall treatment duration [72]. The ability to precisely assess and alter VD using digital software reduces manual errors, leading to more predictable and accurate restorations [71]. The use of interim prostheses, fabricated with digital techniques, can help re-establish VD, followed by the accurate creation of definitive prostheses, so the digital approach enhances consistency and standardization in treatment protocols [78].

Future Directions

The trajectory of digital VD determination points towards exciting future trends and research areas.

Advanced digital fabrication: Future advances, including the application of 4D printing technology, will lead to the production of dentures capable of accommodating various mouth movements, offering enhanced functionality and fit [79].

Enhanced AI integration: AI is expected to play an increasingly prominent role, advancing its ability to gather, analyze, and structure patient data to provide highly personalized and patient-centered dental care [72]. Further research is needed to broaden AI applications beyond diagnostics into treatment planning and implementation for VD.

## Conclusions

Determining the VDO remains a complex clinical challenge, with no single method being universally applicable to all cases. Traditional approaches, such as facial measurements and physiological techniques, offer valuable clinical guidance due to their accessibility and practicality; however, they often lack the precision and reproducibility required in complex situations.

Recent advances in digital technologies, including 3D facial analysis, imaging, and CAD systems, offer greater accuracy and documentation but raise concerns about cost and complexity. The most reliable strategy combines conventional and modern methods, tailored to the individual patient's needs and clinical circumstances. Future research should focus on developing standardized protocols, validating digital tools across populations, and exploring artificial intelligence applications to enhance accuracy, outcomes, and patient satisfaction in prosthodontic practice.
